# Response of cotton growth, yield, and water and nitrogen use efficiency to nitrogen application rate and ionized brackish water irrigation under film-mulched drip fertigation

**DOI:** 10.3389/fpls.2024.1361202

**Published:** 2024-03-28

**Authors:** Kai Wei, Quanjiu Wang, Mingjiang Deng, Shudong Lin, Yi Guo

**Affiliations:** State Key Laboratory of Eco-hydraulics in Northwest Arid Region, Xi’an University of Technology, Xi’an, China

**Keywords:** cotton, ionized brackish water, plant height, shoot dry matter, water-nitrogen use efficiency

## Abstract

**Introduction:**

The presence of brackish water resources is significant in addressing the scarcity of freshwater resources, particularly in the Xinjiang region. Studies focused on reducing adverse effect of brackish water irrigation based on using ionized brackish water, as well as on investigating its effects on fibre and oil plant production processes, remain incipient in the literature. Some benefits of this technique are the optimization of the quality and quantity of irrigation water, economy of water absorbed by the plants, improvement in the vegetative growth and productivity compared to irrigation using conventional brackish water. Thus, the aim of the current study is to assess the effect of different nitrogen application rates on soil water and salinity, cotton growth and water and nitrogen use efficiency.

**Methods:**

The experimental design consisted of completely randomized design with two water types (ionized and non-ionized) and six nitrogen application rates with four replications.

**Results:**

Irrigation conducted with ionized brackish water and different nitrogen application rates had significant effect on the plant height, leaf area index, shoot dry matter, boll number per plant and chlorophyll content. The study also demonstrated significant effects of ionized brackish water on soil water content and soil salinity accumulation. The highest cotton production was achieved with the use of 350 kg·ha^-1^ of ionized brackish water for irrigation, resulting in an average increase of 11.5% compared to the use of non-ionized brackish water. The nitrogen application exhibits a quadratic relationship with nitrogen agronomic use efficiency and apparent nitrogen use efficiency, while it shows a liner relationship with nitrogen physiological use efficiency and nitrogen partial productivity. After taking into account soil salinity, cotton yield, water and nitrogen use efficiency, the optimal nitrogen application rate for ionized brackish water was determined to be 300 kg·ha^^-1^^.

**Discussion:**

It is hoped that this study can contribute to improving water management, reducing the environmental impact without implying great costs for the producer.

## 1 Introduction

With the increasing global population and improving living standards, people’s demand for agricultural products has increased dramatically (Ren et al., 2019, 2022). However, agricultural production requires many materials, especially fertilizer input, which leads to the continuous reduction of available resources and the deterioration of the ecological environment (Gong et al., 2011; Fan et al., 2019). Therefore, it is a major challenge to obtain a higher yield at a lower cost (including economically and environmentally). Low fertilizer inputs are crucial for efficient resource utilization, cleaner crop production, and achieving the goal of peak carbon emission and carbon neutrality (Chen et al., 2014; Gerten et al., 2020; Van Dijk et al., 2021).

Agriculture has become the principal freshwater consumer, accounting for 70% of the global freshwater withdrawals (Foley et al., 2011). In the face of the population growth and good-quality water scarcity (Rodell et al., 2018), a lot of scientists recommend an irrigation with marginal water, i.e., brackish or saline water, for alleviating the pressure imposed from agricultural production on water needs (Skaggs et al., 2014; Assouline et al., 2015; Li and Ren, 2021). In the recent three decades, brackish water has been increasingly applied in many regions, especially in arid and semi-arid areas (Mehta et al., 2000; Yang et al., 2020). Brackish water irrigation can result in salt stress to plants, physiological drought, reduced soil oxygen content, anaerobic respiration by roots, and accumulation of toxic substances (Bouksila et al., 2013). Excess salt affects root water absorption, photosynthesis, and transpiration. This leads to growth inhibition of roots, stems, leaves, and other organs and reduces dry matter production, ultimately leading to a reduction in crop yield (Parida et al., 2004). Owing to the adverse effects on soil properties and plant growth and productivity by irrigation with brackish water, the search continues for more efficient irrigation methods that minimize waste, reduce salt stress, and maintain crop productivity. Ionized water treatment has shown promising potential in saving water resource and promoting agricultural productivity that will be of significant importance in the near future. Wang et al. (2016) reported the ionized treatment of water decreases water surface tension. A reduction in surface tension can enhance the hydration ability of water molecules and ions as well as the mineral salt dissolving capacity. Zhao et al. (2021) demonstrated that irrigation with ionized fresh water improves plant height and yield of winter wheat. Thus, the ionized water treatment can lead to improvements in terms of cleaner and sustainable production.

Cotton (*Gossypium hirsutum* L.) is the most important oil and fiber crop worldwide (Li et al., 2022). Xinjiang, China, is the largest irrigated cotton-producing arid region in the world. It contributed more than 19% to the global production from only 7.8% of the worldwide cotton sown area (Shi et al., 2022). Different nitrogen application rates will affect cotton’s nitrogen absorption (Sun et al., 2023). The nitrogen fertilizer application rate will significantly affect the plant height, main stem nodes, number of bolls per plant, boll weight, and seed cotton yield (Munir et al., 2015). Reductions in growth combined with increased fruit shed under severe N deficiency lead to a decrease in final boll number per plant and end-of-season lint yield (Bondada and Oosterhuis, 2001). Conversely, high N application rates can produce excessive vegetative growth, poor fruit retention at lower mainstem nodes, and delayed crop maturity (Boquet and Breitenbeck, 2000). It was also reported that nitrogen fertilization improves the salinity tolerance of cotton plants, because N played both nutritional and osmotic roles in saline conditions (Ding et al., 2010). However, N management in cotton is particularly difficult due to problems with either excessive or inadequate rates or influence of abiotic stresses like drought of salinity (Rinehardt et al., 2004; Dong et al., 2012). In recent years, fertilizer management in salt-affected cotton fields has attracted a number of interests. Chen et al. (2010) studied the influence of different N fertilization rates and soil salinity levels on the growth and nitrogen uptake of potted cotton plants. They found that cotton growth was significantly affected by the interaction of soil salinity and N but not by N alone. When using saline water for irrigation, excessive nitrogen application would cause more alkaline cations in the soils, increase soil salinity, and inhibit the absorption of nitrogen by roots and thus reduce crop yields (Han et al., 2015). Proper management of N fertilizer is especially important in saline soils where N application might reduce the adverse effects of salinity on plant growth and yield (Hou et al., 2009).

Because of the arid conditions and limited water resources in southern Xinjiang, where much of the region is situated on the desert periphery, saline groundwater (with concentrations ranging from 1 g·L^−1^ to 12 g·L^−1^) was extensively employed as a substitute for fresh water in cotton cultivation. We hypothesize that the use of water treated with an ionized system can increase cotton growth, improve water use efficiency (WUE) and nitrogen use efficiency (NUE), and reduce soil salinity accumulation. However, there is little information available in the literature about cotton irrigation with ionized treated water. Thus, the aim of the current study was to investigate the effect of different nitrogen application rates on soil water and salinity, cotton growth, and WUE and NUE under ionized and non-ionized brackish water irrigation.

## 2 Materials and methods

### 2.1 Experimental site and cultivar

**Figure 1:**
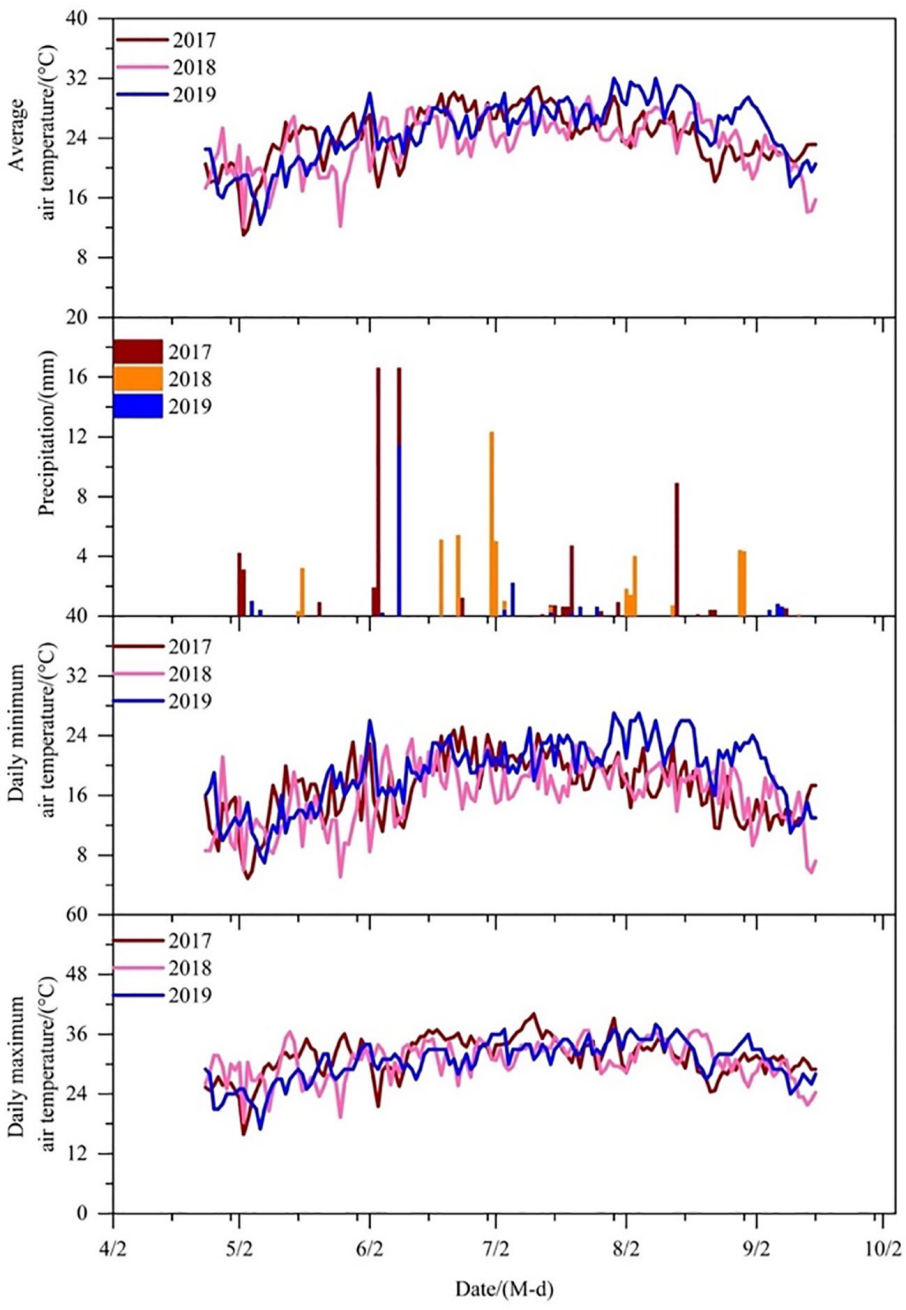
Daily air temperature and precipitation during the cotton growing seasons in 2017, 2018, and 2019.

The 3-year (2017, 2018, and 2019) field trials were conducted at the Experiment Station of Bazhou Irrigation (41°45’20.24”N, 86°8’51.16”E; altitude 901 m) in Korla City, Xinjiang Province, Northwest China. The physical characteristics of the soil layer from 0 to 100 cm are depicted in **Table 1**. According to USDA-NRCS (2020), the soil in the years 2017 and 2018 was categorized as sandy loam. It had a pH level of 8.75, a total organic matter content of 76.8 mg·kg^−1^, a total nitrogen content of 3.92 mg·kg^−1^, an available phosphorus content of 31.1 mg·kg^−1^, and an available potassium content of 72.0 mg·kg^−1^. In 2019, the soil was categorized as sandy with a pH level of 8.82. It contained 89.1 mg·kg^−1^ of total organic matter, 4.87 mg·kg^−1^ of total nitrogen, 30.8 mg·kg^−1^ of available phosphorus, and 79.5 mg·kg^−1^ of available potassium. The groundwater depth is below 7 m. The electronic conductivity (EC) of groundwater is 2.73–2.95 ms·cm^−1^. **Table 2** provides the chemical characteristics of groundwater, with a total dissolved solids concentration of 2.2 g·L^−1^. An automatic weather station was installed in the experimental area to record the daily climatic information, which encompassed precipitation, air temperature, solar radiation, wind speed, relative humidity, and air pressure. According to the weather data, the seasonal precipitation during 2017, 2018, and 2019 cotton seasons was 64.4, 49.8, and 19 mm, respectively. **Figure 1** displays the daily values for the mean air temperature, lowest air temperature, and maximum air temperature, highest air temperature, and rainfall for cotton seasons.

**Table 1:** Physical properties of the soil in the experimental site.

	Soil layer (cm)	Soil texture			Soil texture	Bulk density (g cm^−3^)	*θ*_WP_ (cm^3^ cm^-3^)	*θ*_FC_ (cm^3^ cm^-3^)	*θ*_s_ (cm^3^ cm^-3^)
		Sand (%)	Silt (%)	Clay (%)					
2017 2018	0–20	43.2	52.8	4.0	Silt loam	1.52	0.0309	0.206	0.3319
	20–40	64.0	33.1	2.9	Sandy loam	1.52	0.0297	0.288	0.3502
	40–60	64.5	32.6	2.9	Sandy loam	1.54	0.0296	0.211	0.3466
	60–80	73.9	24.0	2.1	Loamy sand	1.52	0.0328	0.233	0.3630
	80–100	83.6	15.1	1.3	Loamy sand	1.63	0.0379	0.214	0.3425
2019	0–20	83.1	15.3	1.6	Loamy sand	1.47	0.0391	0.250	0.3862
	20–40	89.5	9.7	0.8	Sandy	1.64	0.0426	0.170	0.3412
	40–60	88.6	10.5	0.9	Sandy	1.54	0.0430	0.198	0.3699
	60–80	85.0	13.3	1.7	Loamy sand	1.53	0.0406	0.207	0.3721
	80–100	82.0	16.2	1.8	Loamy sand	1.55	0.0380	0.212	0.3643

**Table 2:** The chemical properties of groundwater during cotton growing season in 2017–2019.

Properties	pH	HCO^3−^ (g·L^−1^)	Cl^−^ (g·L^−1^)	SO_4_^2−^ (g·L^−1^)	Ca^2+^ (g·L^−1^)	Mg^2+^ (g·L^−1^)	K^+^ (g·L^−1^)	Na^+^ (g·L^−1^)
Value	7.38	0.401	0.335	1.110	0.227	0.149	0.029	0.369

### 2.2 Experimental treatments and design

**Figure 2:**
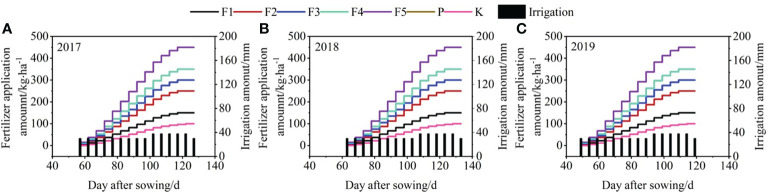
Schedule of irrigation and fertilizer during the cotton growing seasons in 2017 **(A)**, 2018 **(B)**, and 2019 **(C)**.

Field experiments utilized a drip irrigation system with film mulch. The study included six nitrogen levels (0 kg·ha^−1^, 150 kg·ha^−1^, 250 kg·ha^−1^, 300 kg·ha^−1^, 350 kg·ha^−1^, and 450 kg·ha^−1^) and two types of water (non-ionized brackish water and ionized brackish water). N fertilizers used in the experiments were urea (N ≥ 46%). This resulted in the NIF0, NIF1, NIF2, NIF3, NIF4, and NIF5 and IF0, IF1, IF2, IF3, IF4, and IF5 treatments, respectively. The 12 treatments were replicated four times in a randomized block design. **Figure 2** reports the irrigation and fertilizer application schedule for the 2017, 2018, and 2019 cotton seasons in Xinjiang.

**Figure 3:**
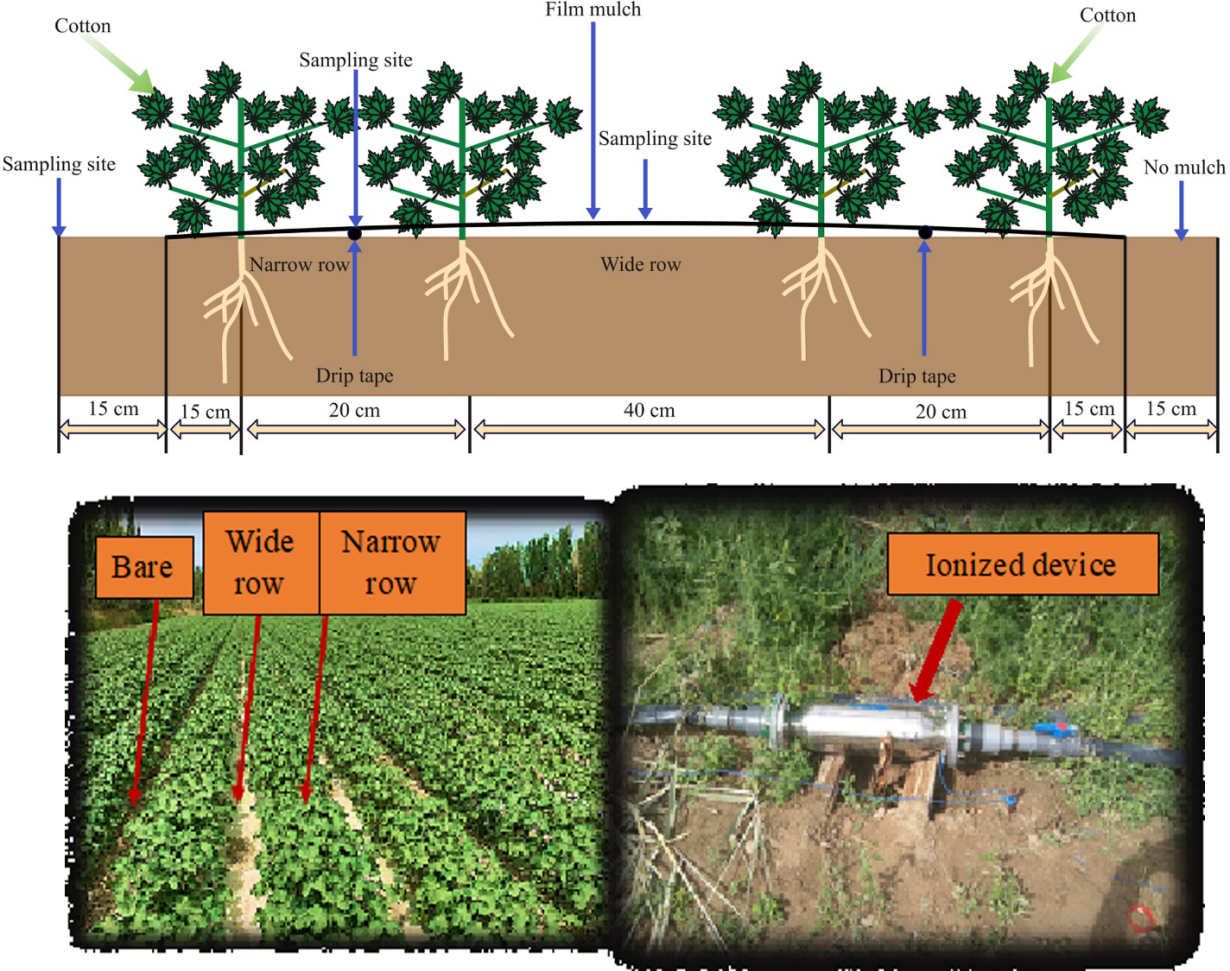
Drip irrigation system arrangement.

Groundwater was employed as the non-ionized brackish water, while the ionized brackish water was taken as groundwater treated via the irrigation system (**Figure 3**). Some properties such as electrical conductivity, pH, total dissolved solids, and surface tension of non-ionized brackish water and ionized brackish water were measured and are listed in **Table 3**.

**Table 3:** Properties of non-ionized brackish water and ionized brackish water.

Treatment	Properties			
	Surface tension	EC	pH	TDS
Non-ionized brackish water	72.0	2.95	7.38	2.2
Ionized brackish water	65.8	2.93	7.27	2.2
Sig	*p<* 0.01	*p* > 0.01	*p* > 0.01	*p* > 0.01

EC is electrical conductivity; TDS is total dissolved solids; Sig is two-tailed *p*-value.

Irrigation was performed using a 16-mm-diameter drip line. The average emitter spacing was 30 cm. Water meters and ball valves were installed to control the amount of water applied to each plot. Each field plot was 5.6 m wide and 10 m long. The discharge rate for each drip emitter was 2.0 L·h^−1^.

Urea (N ≥ 46%) and potassium dihydrogen phosphate (KH_2_PO_4_ ≥ 99.5%) fertilizers were applied 14 times during the cotton growth stage. Differential pressure barrels with a 15-L capacity were used for the fertilizers. One barrel was placed in each experimental plot.

The cotton (*Gossypium hirsutum* L. Xinluzhong) was sowed on 22 April 2017, 24 April 2018, and 28 April 2019. Cotton was planted following the cultivation mode of plastic film mulching and short rod dense planting (**Figure 3**). The narrow–wide–narrow row configuration of the system was set up with measurements of 20 cm + 40 cm + 20 cm. Two driplines were installed for four rows under a 1.1-m-wide film.

### 2.3 Measurements and calculations

#### 2.3.1 Soil water and salt

To determine the water content and salinity of the soil, samples were collected at intervals of 10 cm from 0 to 40 cm and at intervals of 20 cm from 40 cm to 100 cm. This was done using a 5-cm-diameter auger in the middle of wide, narrow, and bare strips. Samples were gathered during the final stages of cotton’s main growth, just prior to irrigation and harvest. After each sampling, the experimental error was minimized by refilling all auger holes with soil. To determine the gravimetric soil water content, the soil samples were weighed, subjected to drying in a fan-assisted oven at a temperature of 105˚C for a duration of 24 h, and then reweighed. The volumetric soil water content was calculated by multiplying the gravimetric soil water content with the average bulk density of the soil profile at a depth of 100 cm. At a temperature of 25°C, the electrical conductivity of a 1:5 soil water extract (EC_1:5_) was measured using a DDS-307 conductivity meter. By applying a linear relationship (SC = 3.946*EC_1:5_, *R* ^2^ = 0.995, *n* = 30), the soil salt content (SC, g·kg^−1^) can be derived from the value EC_1:5_ obtained from each soil sample. The narrow soil strip’s soil salt accumulation was determined by subtracting the salinity content at the beginning of the growth stage from the salinity content at the end. Positive soil salt accumulation indicates the accumulation of salt in the soil, which negative accumulation suggests desalination has taken place.

#### 2.3.2 Crop evapotranspiration

Crop evapotranspiration (water consumption) was determined via the water balance equation as follows (Zhou et al., 2019):


(1) $$ET_{\text{c}}=P+I+G+\Delta W-R_{0}-F$$

where *ET*_c_ represents crop evapotranspiration (mm), *P* represents the precipitation during the growing period (mm), *I* represents irrigation, *G* represents groundwater recharge (mm), Δ*W* represents the change in soil water storage in the 0–100 cm soil layer from sowing to maturity (mm), *R*_0_ represents surface runoff (mm), and *F* represents deep percolation (mm). Since the water level in the experimental area was below 7 m, *G*, *R*_0_, and *F* were considered insignificant, leading to the insignificance of Equation 1. Equation 1 transforms into Equation 2:


(2) $${\text{ET}}_{\text{c}}=\pm \Delta W+P+I$$

Weights were assigned as proportions of the strip widths at different locations, and Δ*W* was calculated as Equation 3:


(3) $$\Delta W=1000\left(\frac{3}{14}\Delta \theta_{bare}+\frac{1}{2}\Delta \theta_{narrow}+\frac{2}{7}\Delta \theta_{wide}\right)$$

where Δθ_bare_, Δθ_narrow_, and Δθ_wide_ are the difference in volumetric soil water content for the bare, narrow, and wide soil strips between late seedling stage and harvest in 100 cm soil profile (cm^3^·cm^−3^), respectively.

#### 2.3.3 Leaf area index

During the seedling, bud, flowering, boll development, and boll-opening stages, four plants were chosen at random from every plot. To ascertain the leaf area, the dimensions of every leaf on the plants were measured using a tape measure (Jha et al., 2019). The calculation of the plant’s leaf area was performed in the following manner (Equation 4):


(4) $$\mathrm{LA}=\sum_{\text{i}}^{\text{n}}{\text{A}}_{\text{i}}*{\text{B}}_{\text{i}}*0.703$$

where LA represents the leaf area per plant (cm^2^), *A*_i_ (cm) and *B*_i_ (cm) represent the length and width of a leaf of cotton, *n* represents the number of leaves per plant of cotton, and 0.703 is the cotton leaf area correction factor (Wei et al., 2022).

The leaf area index (LAI) was then obtained as follows (Equation 5) (Wang et al., 2018; Wang X. et al., 2020):


(5) $${\text{LAI=LA}}_{\text{T}}{\text{/S}}_{\text{O}}$$

where LA_T_ represents the total area of the cotton leaves (cm^2^) and S_O_ represents the occupied land area (cm^2^).

#### 2.3.4 Shoot dry matter

At the seedling stage, bud stage, flowering phase, boll development stage, and boll-opening stage, four plants were randomly selected from each plot. The leaves, stem, squares, flowers, and bolls were placed into an oven at 105°C for 30 min and then dried at 75°C to constant weight and weighed. The average weight of four plants multiplied by the planting density per hectare represented the dry matter accumulation per hectare.

#### 2.3.5 Relative chlorophyll content (SPAD value)

The measurement of SPAD was conducted using a chlorophyll meter called SPAD-502 Plus. This was done on a functional cotton leaf that was young and fully expanded. The leaf chosen was the fourth one below the main stem terminal before the plant was topped, and the second leaf from the top after topping. In every plot, 15 leaves were chosen at random and two measurements were taken on each leaf, one on either side of the midrib.

#### 2.3.6 Cotton yield

The measurement of cotton production was conducted when 90% of the cotton bolls had opened. In each of the experimental plots, three sampling grids measuring 2.33 m × 2 m were established. Along these grids, a total of bolls were collected and their weights were measured (Wang et al., 2020).

#### 2.3.7 Water use efficiency

WUE (Equation 6) was calculated as follows (Gang et al., 2019):


(6) $${\text{WUE=Y/ET}}_{\text{c}}$$

where *Y* represents the cotton yield (kg ha^−1^) and *ET*_c_ represents the crop evapotranspiration (mm).

#### 2.3.8 Nitrogen use efficiency

Partial productivity of nitrogen (NPFP) (Equation 7) was calculated by Dai et al. (2023).


(7) $$NPFP=Y/F_{t}$$

where *Y* is the cotton yield (kg ha^−1^) and *F*_t_ is the amount of fertilizer N applied (kg ha^−1^).

Agronomic nitrogen use efficiency (aNUE) (Equation 8) is defined as the increase in cotton yield per unit of fertilizer N applied (Zhang et al., 2012):


(8) $$\text{aNUE}=\left(\text{Y}-{\text{Y}}_{0}\right)/{\text{F}}_{\text{t}}$$

where *Y* is the cotton yield (kg ha^−1^), *Y*_0_ is the cotton yield of unfertilized treatment (kg ha^−1^), and *F*_t_ is the amount of fertilizer N applied (kg ha^−1^).

Nitrogen apparent recovery efficiency (ARE_N_) (Equation 9) is calculated by Xu et al. (2023).


(9) $$ARE_{N}=\left(NU_{A}-NU_{0}\right)/F_{N}$$

Physiological nitrogen use efficiency (pNUE) (Equation 10) is defined as the increase in cotton yield per unit of increased N uptake as reported in Isfan (1990).


(10) $$pNUE=\frac{Y-Y_{0}}{NU_{A}-NU_{0}}$$

where *F*_N_ represents the amount of fertilizer N applied (kg ha^−1^), *NU*_A_ represents total N uptake of N-fertilizer plots (kg ha^−1^), and *NU*_0_ represents total N uptake of zero N plots (kg ha^−1^).

The total nitrogen uptake (NU) (Equation 11) by crops is calculated by Li et al. (2021).


(11) NU=NC×SDM

where *NC* represents plant nitrogen content (%) and *SDM* represents shoot dry matter weight (kg ha^−1^).

#### 2.3.9 Relationship between the cotton yield, shoot dry matter, WUE, and nitrogen application rates

A quadratic function was used to fit the relationship among the cotton yield, shoot dry matter, WUE, and nitrogen application rates. The quadratic equation (Equation 12) can be expressed as:


(12) $${\text{Y=aX}}^{2}\text{+ bX+c}$$

where *Y* represents the cotton yield (kg ha^−1^), *X* represents the nitrogen application rates (kg ha^−1^), and *a*, *b*, and *c* are coefficient.

### 2.4 Data analysis

SPSS statistics 22 software was employed to perform analysis of variance (ANOVA). The average of four replicates was displayed for all indicators. The significant difference among all treatments was determined by least significant difference (LSD) at the *p<* 0.05 level. Origin 2021, Microsoft PowerPoint 2020, and Microsoft Excel 2020 were used to create figures and analyze data, respectively.

## 3 Results

### 3.1 Plant height

**Figure 4:**
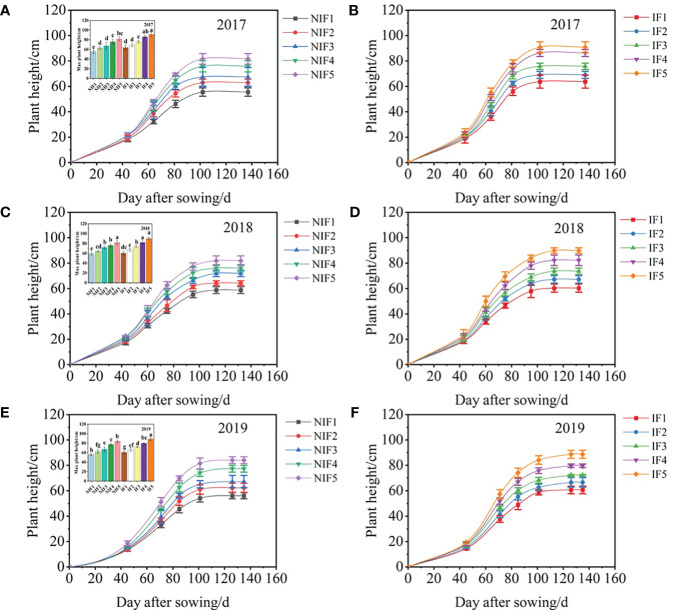
Effects of nitrogen application rates and water type on the cotton plant height with the days after sowing (DAS) for 2017–2019 cotton growing seasons **(A–F)**. Data are the mean value of the four replicates. Errors bars denote mean standard errors. Different letters above the bars indicate significant differences among treatments at *p* < 0.05.

In 2017, 2018, and 2019, the results of this study exhibited significant impacts of nitrogen application rates and water types on the cotton plant height (*p<* 0.05, **Figure 4**). During 2017, 2018, and 2019, there was a trend of rapid growth followed by stabilization in cotton plant height, with maximum height being achieved at 81, 95, and 101 days after sowing, respectively. Plant height increased with nitrogen application rates in both the ionized and non-ionized treatment, with treatment IF5 maximizing plant height in 2017, 2018, and 2019. Ionized treatments exhibit a promotional effect on the maximum height of cotton plants in comparison to the non-ionized treatments.

### 3.2 Leaf area index

**Figure 5:**
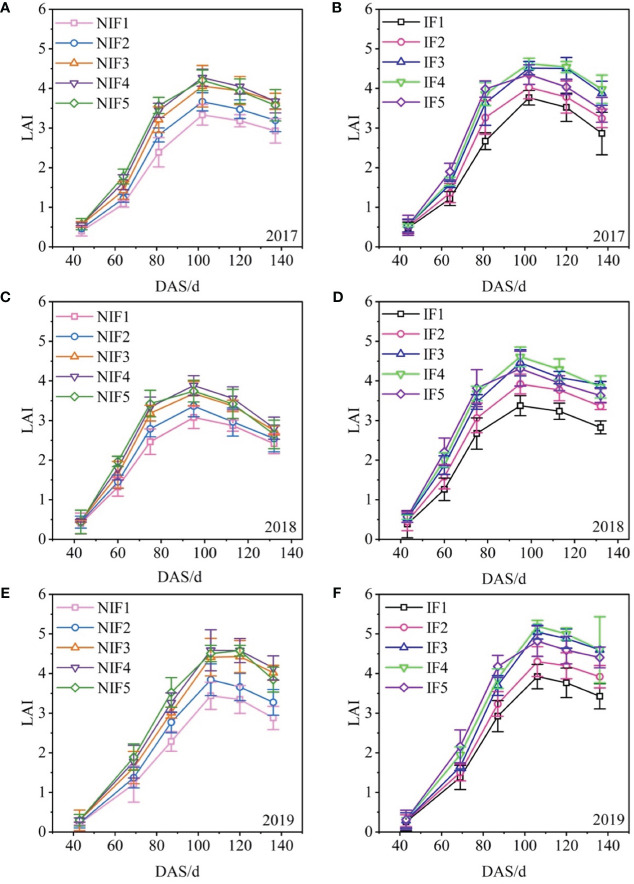
Effect of water types and nitrogen application rates on the leaf area for 2017–2019 cotton growing seasons (A-F). Data are the mean value of the four replicates. Errors bars denote mean standard errors.

LAI varied among water types and nitrogen application rates, but all showed an opening down unimodal curve as days after sowing (**Figure 5**). A significant effect was exerted by water types and nitrogen application rates on LAI (*p<* 0.05). The LAI reached the peak at approximately 102, 95, and 106 days after sowing in 2017, 2018, and 2019, respectively. The peak value of LAI ranged from 3.3 to 4.6 in 2017, from 3.1 to 4.6 in 2018, and from 3.4 to 5.2 in 2019. Differences between nitrogen application rates and water type treatment of the LAI were small at the earlier stages of growth and then gradually increased. During the whole growth period, the LAI of ionized treatments was always higher than non-ionized treatments, by up to 5.2. Three-year results showed that the peak LAI of ionized treatments were 10.1%–15.0% higher than that of the non-ionized treatment.

### 3.3 Shoot dry matter

**Figure 6:**
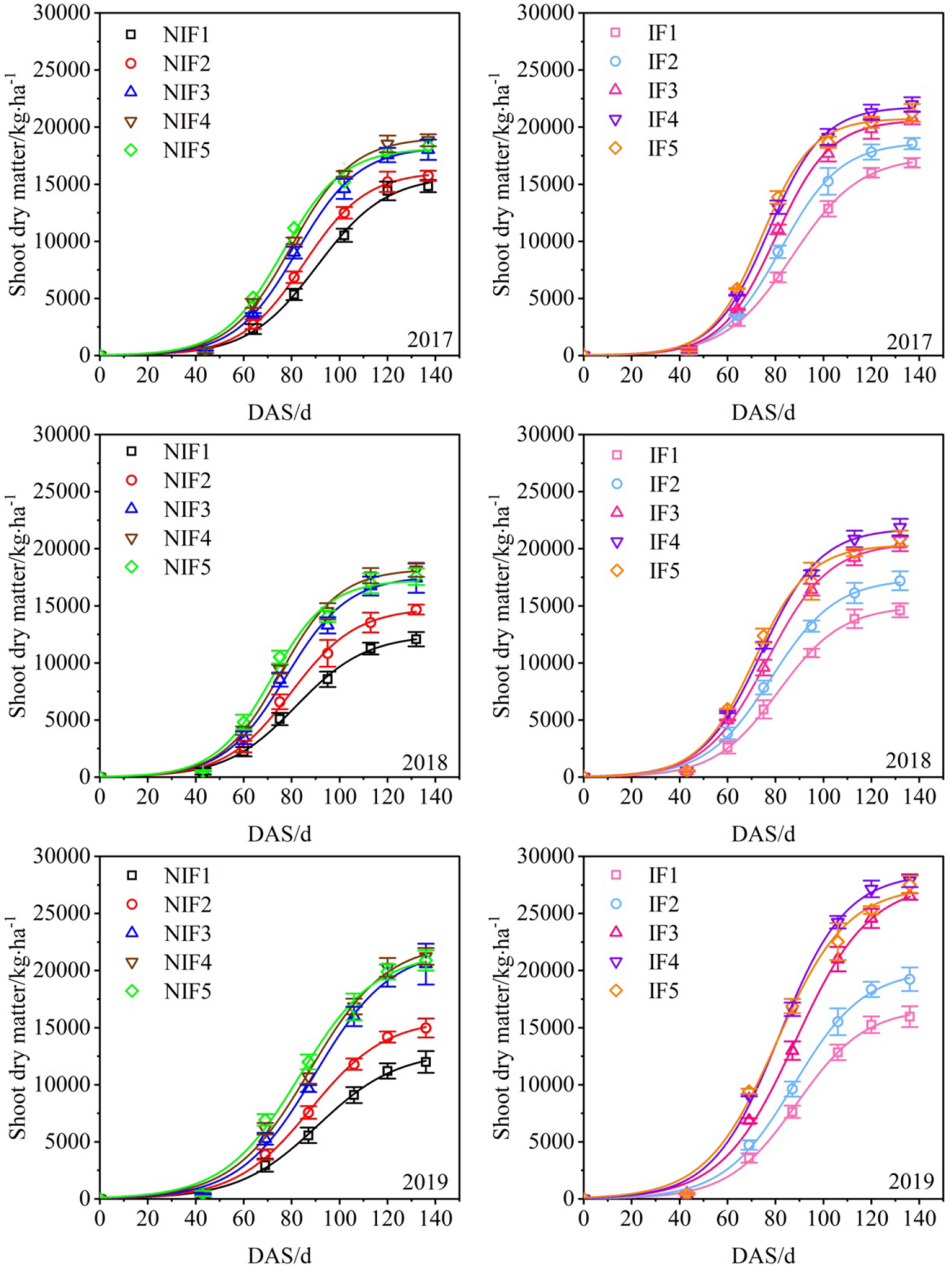
Effect of water types and nitrogen application rates on the shoot dry matter for 2017–2019 cotton growing seasons. Data are the mean value of the four replicates. Errors bars denote mean standard errors.

The shoot dry matter of cotton was different in water types and nitrogen application rates. The shoot dry matter of ionized treatments was higher than that of non-ionized treatments (**Figure 6**). The shoot dry matter of IF4 was the highest in 3 years, and the highest was 27,870 kg·ha^−1^. The shoot dry matter of NIF1 was the lowest in 3 years, which was 12,003 kg·ha^−1^.

### 3.4 Relative chlorophyll content (SPAD)

**Figure 7:**
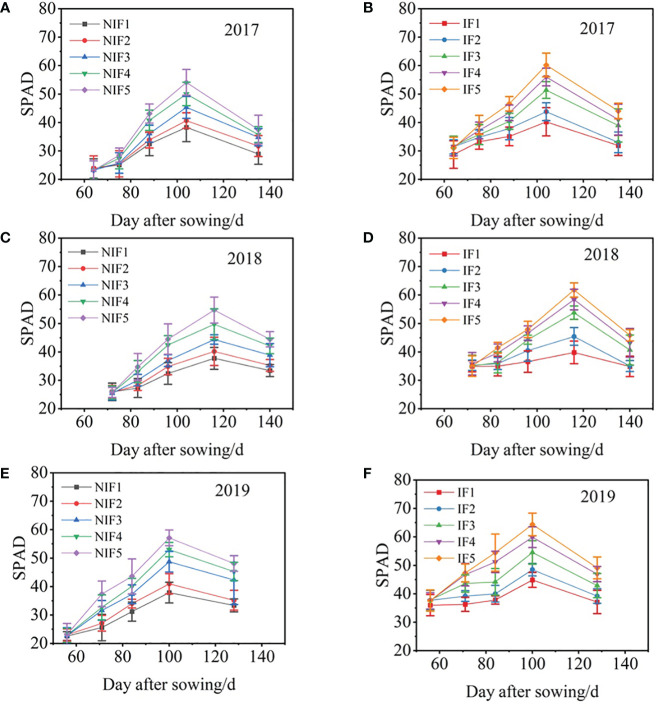
Changes of SPAD with day after sowing for 2017–2019 cotton growing seasons **(A–F)**. Data are the mean value of the four replicates. Errors bars denote mean standard errors.

Relative chlorophyll content changes in the different nitrogen application rates and water types described a unimodal curve during the growth period (**Figure 7**). The SPAD peak values of ionized treatments were generally higher than those of the non-ionized treatments. Furthermore, for the peak value of SPAD, IF5 was the highest and NIF1 was the lowest. Fertilizer had a significant positive effect on relative chlorophyll content. With the increase of nitrogen application rate, the SPAD increased. The results showed that the peak relative chlorophyll content of ionized treatments was 5.0%–13.4%, 5.5%–21.3%, and 11.9%–18.5% higher than that of the non-ionized treatment in 2017, 2018, and 2019, respectively.

### 3.5 Cotton yield and water use efficiency

The nitrogen application rates and water types had a significant effect on cotton yield (*p<* 0.05) (**Table 4**). During the years 2017, 2018, and 2019, the highest yield was observed when using non-ionized brackish water treatment NIF4, with a nitrogen application rate of 350 kg·ha^−1^, while the lowest yield was obtained with NIF0. The highest yield was observed when subjected to ionized brackish water treatment IF4, while the lowest yield was recorded under IF0. Fertilizer had a significant positive effect on cotton yield. With the increase of nitrogen application rate, the cotton yield firstly increased and then decreased. The results showed that the yield of ionized treatments was 11.3%–21.2%, 3.9%–13.4%, and 12.0%–29.2% higher than that of the non-ionized treatment in 2017, 2018, and 2019, respectively.

**Table 4:** Effects of non-ionized brackish water and ionized brackish water nitrogen level on cotton yield and water and fertilizer use efficiency in 2017, 2018, and 2019.

Year	Treatment	Yield (kg·ha^−1^)	*P* (mm)	*I* (mm)	Δ*W* (mm)	*ET*_c_ (mm)	WUE (kg·ha^−1^·mm)
2017	NIF0	2,451.1f	64.4	487.5	–	–	–
	NIF1	4,186.8e	64.4	487.5	7.9g	559.8g	7.5e
	NIF2	5,543.6d	64.4	487.5	11.5f	563.4f	9.8d
	NIF3	6,413.9c	64.4	487.5	19.1e	570.9e	11.2c
	NIF4	6,991.9bc	64.4	487.5	28.6bc	580.5bc	12.0bc
	NIF5	6,496.6c	64.4	487.5	27.0bc	578.9bc	11.2c
	IF0	2,851.1f	64.4	487.5	–	–	–
	IF1	4,657.9e	64.4	487.5	18.2e	570.1e	8.2e
	IF2	6,468.6c	64.4	487.5	23.3d	575.2d	11.2c
	IF3	7,771.4a	64.4	487.5	29.2b	581.1b	13.4a
	IF4	7,820.3a	64.4	487.5	35.4a	587.3a	13.3a
	IF5	7,406.7ab	64.4	487.5	26.3c	578.2c	12.8ab
2018	NIF0	2,312.5f	64.4	487.5	–	–	–
	NIF1	3,854.9e	49.8	487.5	23.6g	560.9g	6.9e
	NIF2	5,332.7d	49.8	487.5	31.9f	569.2f	9.4d
	NIF3	6,169.4bc	49.8	487.5	37.1e	574.4e	10.7bc
	NIF4	6,827.1a	49.8	487.5	44.0c	581.3c	11.7a
	NIF5	6,338.8b	49.8	487.5	41.6cd	578.9cd	10.9b
	IF0	2,562.5f	64.4	487.5	–	–	–
	IF1	4,125.6e	49.8	487.5	32.4f	569.7f	7.2e
	IF2	5,845.0c	49.8	487.5	39.8de	577.1de	10.1c
	IF3	6,997.9a	49.8	487.5	47.6b	584.9b	12.0a
	IF4	7,094.3a	49.8	487.5	56.4a	593.7a	11.9a
	IF5	6,955.9a	49.8	487.5	50.6b	587.9b	11.9a
2019	NIF0	2,376.0f	64.4	487.5	–	–	–
	NIF1	4,124.1e	19	487.5	42.3f	548.8f	7.5e
	NIF2	5,720.2d	19	487.5	48.9e	555.4e	10.3cd
	NIF3	6,464.6c	19	487.5	57.8d	564.3d	11.5bc
	NIF4	7,080.5b	19	487.5	62.9c	569.4c	12.4b
	NIF5	6,480.5bc	19	487.5	60.3cd	566.8cd	11.4bc
	IF0	2,783.5f	64.4	487.5	–	–	–
	IF1	4,619.8e	19	487.5	51.2e	557.7e	8.3de
	IF2	7,043.6bc	19	487.5	63.1c	569.6c	12.4bc
	IF3	8,352.5a	19	487.5	70.9b	577.4b	14.5a
	IF4	8,408.2a	19	487.5	76.6a	583.1a	14.4a
	IF5	7,903.7a	19	487.5	71.9b	578.4b	13.7a

*P* is precipitation; *I* is irrigation; Δ*W* is change in the soil water storage; *ET*_c_ is crop evapotranspiration; WUE is water use efficiency. Data are mean of the four replicates. The different letters indicate significant differences between treatments at the *p* < 0.05 level according to the LSD test; the same letters are not significantly different at *p* > 0.05 level according to the LSD test.

Nitrogen application rates and water types had a significant effect on WUE (*p<* 0.05) (**Table 4**). IF3 had the highest WUE, while NIF1 had the lowest. The average values over 3 years were 13.3 kg·ha^−1^·mm^−1^ for IF3 and 7.3 kg·ha^−1^·mm^−1^ for NIF1. The WUE of ionized treatments was 9.3%–19.6%, 1.7%–12.1%, and 10.7%–26.1% higher than that of the non-ionized treatment in 2017, 2018, and 2019, respectively.

### 3.6 Relationship between seed cotton yield, shoot dry matter, and water use efficiency and nitrogen application rate

**Figure 8:**
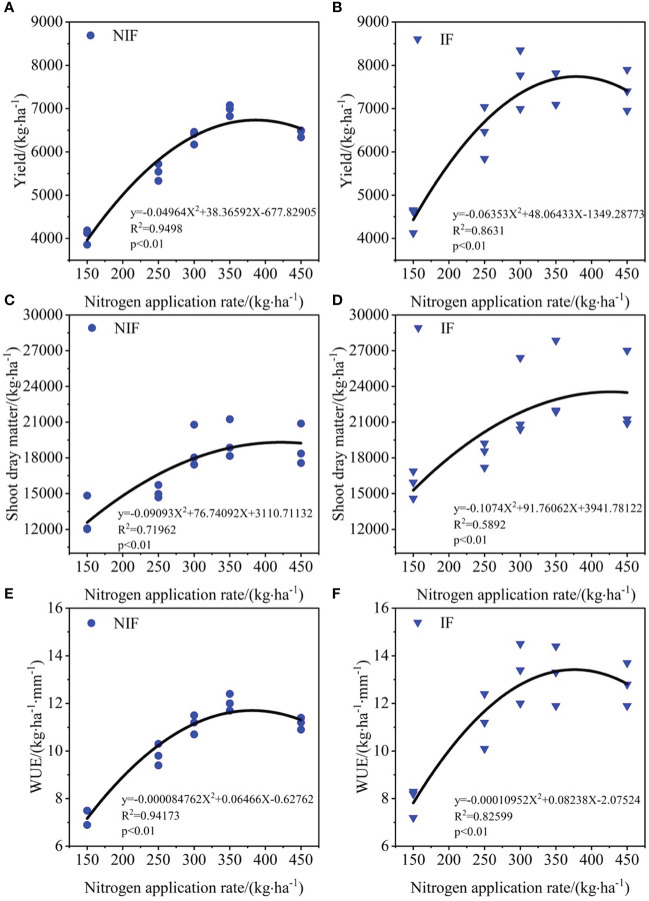
Relationship among nitrogen application rates and seed cotton yield **(A, B)**, shoot dry matter **(C, D)**, and water use efficiency (WUE) **(E, F)**.

The relationship between seed cotton yield, shoot dry matter, WUE, and nitrogen application rate follows a quadratic curve (**Figure 8**).

### 3.7 Soil water content

**Figure 9:**
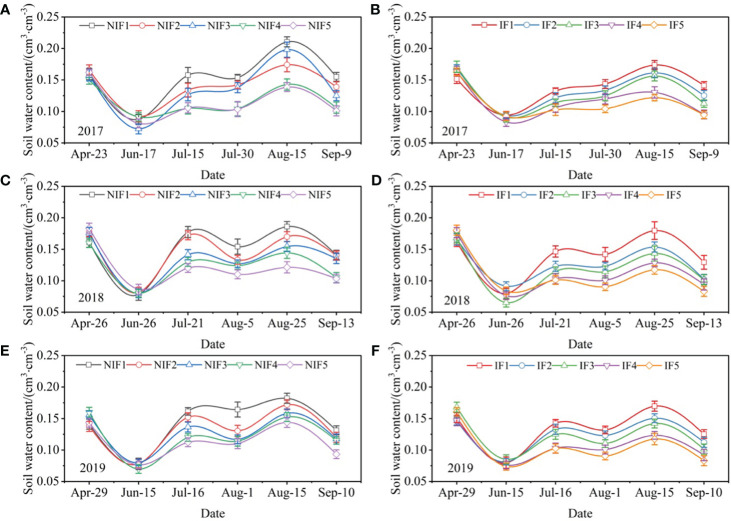
Dynamics of soil volumetric water content in 0–40 cm from the seedling stage to maturity stage of cotton under non-ionized brackish water **(A, C, E)** and ionized brackish water **(B, D, F)** and nitrogen amounts in 2017, 2018, and 2019. Data are the mean value of the four replicates. Errors bars denote mean standard errors.

The changes of the volumetric water content of the soil under ionized and non-ionized treatments are shown in **Figure 9**. Fertilizer had a significant effect on soil water content. With the increase of nitrogen application rate, the soil water content decreased. The soil with ionized brackish water irrigation decreased the soil water content compared to the soil with non-ionized brackish water irrigation. The average soil water content of ionized treatments was 10.5%, 17.4%, and 13.6% lower than that of non-ionized treatments in 2017, 2018, and 2019, respectively.

### 3.8 Soil salinity

To avoid the influences of initial soil salt content and better evaluate the effects of ionized treatments and nitrogen application rates on soil salinity, the salt accumulation from late seedling stage to harvest was analyzed (**Table 5**). Ionized treatments and nitrogen application rate affected soil salt accumulation (*p<* 0.05). Cotton roots were mainly located with the top 0–40 cm of soil under film-mulched drip irrigation. Therefore, the top 0–40 cm soil layer was considered as the main root zone of cotton in the following analysis. The soil salt accumulation of ionized treatments was 59.6%, 65.3%, and 51.8% lower than that of non-ionized treatments in 2017, 2018, and 2019, respectively. The soil salt desalination of the 0–40 cm soil profile was maximized at IF4 in 2017, but no significant differences in soil salt desalination were observed between IF3 and IF4. The soil salt desalination of the 40 cm soil profile was maximized at IF3 in 2018 and 2019, but no significant differences soil salt desalination were observed between IF3 and IF4.

**Table 5:** Soil salt accumulation for all treatments in a narrow strip in the 40-cm and 100-cm soil profile in 2017, 2018, and 2019.

Treatment	Soil salinity accumulation/g·m^−2^					
	2017		2018		2019	
	0–40 cm	0–100 cm	0–40 cm	0–100 cm	0–40 cm	0–100 cm
NIF1	286.2a	235.5f	155.9a	241.9d	192.8a	211.3g
NIF2	130.6bcd	342.5e	91.8abc	369.3c	125.4b	375.3e
NIF3	−261.0f	404.1cde	−236.8e	487.5b	−142.3c	438.8d
NIF4	−176.5e	465.7abc	−91.7d	496.3b	−104.1c	462.1cd
NIF5	165.7bc	496.5a	146.4a	520.4b	114.3b	632.5a
IF1	188.7b	265.4f	110.6ab	323.4c	127.8b	269.6f
IF2	102.5cd	368.5de	76.4c	475.7b	69.9b	436.5d
IF3	−389.9g	420.5bcd	−303.2f	587.0a	−216.9d	518.3bc
IF4	−416.1g	486.3ab	−277.3ef	593.6a	−201.7d	548.7b
IF5	71.1d	512.3a	77.9bc	619.8a	74.8b	664.1a

Different letters within a column indicate significant differences among all treatments, *p*< 0.05. “-” represent soil salt desalination.

### 3.9 Nitrogen use efficiency

In 3 years, nitrogen application rate and water type had significant effects on aNUE, nitrogen apparent recovery efficiency, nitrogen physiological use efficiency, and nitrogen partial factor productivity (*p<* 0.05) (**Table 6**). For the aNUE, IF3 was the highest and NIF5 was the lowest, and the 3-year average values were 16.9 kg·kg^−1^ and 8.8 kg·kg^−1^, respectively. With the increase of the nitrogen application rate, the aNUE firstly increased and then decreased. Average aNUE in the ionized treatment was 13.5%, 26.0%, and 24.3% higher than that of the non-ionized treatment in 2017, 2018, and 2019, respectively. For the nitrogen apparent recovery efficiency, IF3 was the highest and NIF1 was the lowest. With the increase of nitrogen application rate, the aNUE firstly increased and then decreased. pNUE decreased with the increase of nitrogen application rate. In 2017, the highest NPFP for IF1 treatment was 31.1 kg·ha^−1^, followed by 27.5 kg·ha^−1^ in 2018 and 30.8 kg·ha^−1^ in 2019. NPFP decreased with the increase of nitrogen application rate. Average aNUE in the ionized treatment was 14.5%, 8.8%, and 21.4% higher than that of the non-ionized treatment in 2017, 2018, and 2019, respectively.

**Table 6:** Effects of different treatments on agronomic nitrogen use efficiency, nitrogen apparent recovery efficiency, nitrogen physiological use efficiency, and nitrogen partial factor productivity.

Year	Treatment	Agronomic nitrogen use efficiency (aNUE)/(kg/kg)	Nitrogen apparent recovery efficiency (ARE_N_)/(kg/kg)	Nitrogen physiological use efficiency (pNUE)/(kg/kg)	Nitrogen partial factor productivity (NPFP)/(kg/kg)
2017	NIF1	11.6bcd	0.470e	25.40a	27.9b
	NIF2	12.4bcd	0.563de	23.50ab	22.2c
	NIF3	13.2abc	0.598cde	22.15ab	21.4c
	NIF4	13.0abc	0.673cd	19.36ab	20.0c
	NIF5	9.0d	0.503e	18.03ab	14.4d
	IF1	12.0bcd	0.483e	26.41a	31.1a
	IF2	14.5ab	0.750bc	20.00ab	25.9b
	IF3	16.4a	0.903a	18.38ab	25.9b
	IF4	14.2ab	0.883ab	16.30ab	22.3c
	IF5	10.1cd	0.665cd	15.16b	16.5d
2018	NIF1	8.6de	0.363e	24.55ab	25.7b
	NIF2	11.1c	0.495cd	22.55abc	21.3d
	NIF3	12.0 bc	0.595c	20.26bcd	20.6de
	NIF4	12.2bc	0.711b	17.25cd	19.5e
	NIF5	8.4c	0.528cd	15.93d	14.1f
	IF1	12.1bc	0.468d	27.75a	27.5a
	IF2	14.1ab	0.719b	19.64bcd	23.4c
	IF3	15.6a	0.945a	16.52d	23.3c
	IF4	13.7ab	0.937a	15.03d	20.3de
	IF5	10.4cd	0.704b	14.67d	15.6f
2019	NIF1	11.7cd	0.381g	34.12a	27.5b
	NIF2	12.7c	0.518ef	25.98abc	22.2cd
	NIF3	13.6bc	0.697bc	19.46bc	21.5d
	NIF4	13.5c	0.767b	17.54bc	20.2d
	NIF5	9.1d	0.577de	15.87c	14.4f
	IF1	12.2c	0.453fg	28.53ab	30.8a
	IF2	17.0a	0.609cde	28.30ab	28.2b
	IF3	18.6a	0.941a	19.75bc	27.8b
	IF4	16.1ab	0.927a	17.34bc	24.0c
	IF5	11.4cd	0.682bcd	16.75c	17.6e

Data are presented as mean values (*n* = 4); values followed by different lowercase letters indicate significance at *p* < 0.05.

## 4 Discussion

### 4.1 Effects of nitrogen application rates and water type on the plant height, LAI, chlorophyll content, shoot dry matter, and yield

Ionized fresh water significantly improves the plant height, aboveground biomass, and SPAD value of winter wheat (Wang X. et al., 2020). Our results are consistent with previous studies, which also showed that cotton grown with ionized brackish water exhibited greater plant height, LAI, aboveground biomass, and SPAD value compared to cotton grown with non-ionized brackish water. Nitrogen application also has a significant impact on aboveground growth. Increasing nitrogen application led to an increase in plant height, leaf area, and aboveground biomass of crops (Zhang et al., 2021; Qi and Pan, 2022; Snider et al., 2021). Si et al. (2020) conducted experiments on winter wheat and found that increasing nitrogen application enhanced the LAI and aboveground biomass, but these indicators declined when nitrogen was applied excessively. Wang S. et al. (2021) observed that increasing nitrogen application resulted in larger LAI and canopy photosynthetic rate in cotton. This study indicates that nitrogen application has a significant impact on plant height, LAI, and aboveground biomass. Plant height increases with increasing nitrogen application, while the LAI and aboveground biomass initially increase and then decrease with increasing nitrogen application. If the nitrogen application rate surpasses 350 kg·ha^−1^, the LAI and aboveground biomass cease to rise or may even exhibit a decline, suggesting that an excessive amount of nitrogen application could hinder the cotton’s growth.

Nitrogen in the soil can preferably be absorbed and utilized by crops in an inorganic form, and the transformation of nitrogen is closely related to crop uptake. The presence of nitrogen is essential for the production of proteins, nucleic acids, chlorophyll, and other important compounds in crops, while also governing the growth and development processes of crops. Nitrogen is a major component of chloroplasts, enabling plants to carry out photosynthesis and normal metabolism. The nitrogen nutrition level of leaves directly affects their photosynthetic activity, and within a certain range, the photosynthetic rate increases with increasing nitrogen application (Sui et al., 2013; Iqbal et al., 2020). In this study, under both non-ionized brackish water and ionized brackish water, the maximum SPAD value increased with increasing nitrogen application. When nitrogen application was the same, the maximum SPAD value under ionized brackish water was 5.0% to 13.4%, 5.5% to 21.3%, and 11.9% to 18.5% higher than under non-ionized brackish water. The reason why ionized brackish water can increase the SPAD value is mainly because it improves the soil water, salt, and nutrient environment; promotes the nitrogen uptake of cotton; and enhances the functional efficiency of the photosynthetic system.

The appropriate application of nitrogen fertilizer helps improve carbon–nitrogen metabolism. Nitrogen is one of the essential elements required for plant growth and participates in various metabolic processes within plants. Nitrogen application increases the adequacy of nitrogen supply within cotton plants, making it easier for them to absorb and utilize nutrients from soil, thus promoting photosynthesis and respiration. Additionally, nitrogen application can stimulate protein synthesis within cotton, enhancing photosynthesis efficiency and growth rate, consequently increasing carbon content with cotton, optimizing the balance between carbon and nitrogen metabolism, promoting cotton boll development, and thereby increasing the number and weight of cotton bolls per unit area (Stamatiadis et al., 2016). Adequate nitrogen supply promotes increased branching and leaf area in cotton, leading to the accumulation of photosynthetic products and increased cotton boll filling rate and weight, thus boosting yield. However, excessive nitrogen supply can result in excessive cotton growth, increased flower shedding, and reduced cotton boll yield and quality. The impact of various forms of nitrogen (NH_4_^+^ or NO_3_^−^) applied in fertilizer on crop growth can be influenced by other factors such as climate, soil type, rhizosphere pH, and plant species. Chen et al. (2016) found that within the range of 0–375 kg·ha^−1^ of nitrogen application, both the number of cotton bolls and individual boll weight increased with increasing nitrogen application. Nevertheless, in the nitrogen application range of 375–600 kg·ha^−1^, there was a decline in both the quantity of bolls and the weight of each boll as the nitrogen application increased. This study also yielded consistent results. The application of fertilizer resulted in an increase in cotton yield; however, excessive nitrogen application may result in decreased yields (Albornoz and Lietn, 2015; Wang Z. et al., 2021). Similar results were obtained in this study. Studies have shown that either insufficient or excessive nitrogen application can lead to decreased yield (Raphael et al., 2019; Hassanzadehdelouei et al., 2022). The results of this study demonstrate that amount of nitrogen application significantly affects cotton yield and quality. Low nitrogen application limits the accumulation of cotton biomass and plant nitrogen accumulation, thus hindering high yields. Excessive nitrogen application leads to nitrogen wastage and cannot sustain increased yields. Therefore, the appropriate nitrogen application promotes cotton nitrogen absorption, which is consistent with previous research (Ma et al., 2022). The highest production was achieved with a nitrogen application rate of 350 kg·ha^−1^ under both NIF and IF circumstances, and yields started to decrease above 350 kg·ha^−1^. When the nitrogen application rate was the same, the IF treatment increased cotton yield by 11.3% to 21.2%, 3.9% to 13.4%, and 12.0% to 29.2% in 2017, 2018, and 2019 years, respectively. Hou et al. (2021) studied the impact of nitrogen application on cotton yield and concluded that cotton yield was related to nitrogen application in a quadratic polynomial manner, with maximum cotton yield achieved at a nitrogen application rate of 350 kg·ha^−1^, and cotton yield had no significant difference between 350 kg·ha^−1^and 400 kg·ha^−1^.

### 4.2 Effects of nitrogen application rates and water type on soil water and soil salinity

In the 0–40 cm soil layer, under non-ionized brackish irrigation for 2017, 2018, and 2019, the average soil volumetric water content from the budding stage to maturity was 9.5%, 17.4%, and 13.6% higher compared to ionized brackish water irrigation, respectively. Soil volumetric water content gradually decreased with increasing nitrogen application rates. This is because higher nitrogen application rates promote crop growth, resulting in greater aboveground biomass and LAI, which, in turn, requires more water consumption. This observation is consistent with the findings of Kumar et al. (2022). Appropriate nitrogen application rates can reduce salt stress (Ding et al., 2010), promote crop growth, enhance crop nitrogen absorption (Zhang et al., 2018), and mitigate environmental pollution issues caused by excessive nitrogen fertilizer (Liu et al., 2021). Excessive nitrogen fertilizer input leads to the release of a large number protons through nitrification, which further induces an increase in alkaline cations, accelerating soil salinization (Yang et al., 2018). This study found that with increasing nitrogen application rates, the cumulative soil salt content in the 0–40 cm soil layer exhibited a trend of initially decreasing and then increasing, with higher levels observed under NIF irrigation compared to IF; this is because nitrogen application reduces soil pH, increases the dissolution of Ca^2+^ in the soil, and minimizes salt damage by maximizing the competition between Ca^2+^ and Na^+^. Nitrate can balance excess chloride ions and also reduce chloride salinity in the root zone (Dong et al., 2022).

### 4.3 Effects of nitrogen application rates and water type on water use efficiency and nitrogen use efficiency

The previous research demonstrated an increase in WUE with increasing nitrogen application, followed by a decrease, reaching its peak at a nitrogen application rate of 300 kg·ha^−1^ (Chen et al., 2010). Similar results were obtained in this study, showing quadratic relationships between seed cotton yield, aboveground biomass, and WUE with nitrogen application rate.

Increasing nitrogen fertilizer costs and global concern for greenhouse gas emission have resulted in growing interest in improving N use efficiency over the past 20 years (Guo et al., 2022). Understanding how N use efficiency changes with N fertilization rates will assist producers in N management decisions that affect both the profitability and N impact on environment (Rochester, 2011). When evaluating nitrogen fertilizer utilization efficiency, various indicators are typically considered, including aNUE, nitrogen apparent recovery efficiency, pNUE, and partial productivity of nitrogen. aNUE represents the increase in grain yield per unit of nitrogen fertilizer input. Specifically, the nitrogen apparent recovery efficiency refers to the increase in grain yield per unit of nitrogen fertilizer input compared to the accumulation of nitrogen in aboveground plant parts. This concept was introduced for the first time in grain production. Partial productivity of nitrogen combines multiple factors such as soil nutrient levels and fertilizer application rates, making it an important indicator for assessing NUE. NUE is primarily influenced by factors such as crop genotype, farming practices, planting density, fertilizer type, and timing of application. Therefore, optimizing field management practices and nitrogen fertilizer application is a crucial pathway to improving nitrogen fertilizer utilization. In this study, under non-ionized brackish water (NIF) and ionized brackish water (IF) irrigation, nitrogen agronomic use efficiency and apparent use efficiency both showed an initial increase followed by a decrease with increasing nitrogen application. Nitrogen physiological use efficiency and partial productivity of nitrogen decreased with increasing nitrogen application. Nitrogen apparent use efficiency is an indicator of nitrogen absorption potential, reaching its maximum at a nitrogen application rate of 350 kg·ha^−1^ under non-ionized brackish water irrigation and at 300 kg·ha^−1^ under ionized brackish water irrigation, promoting nutrient absorption by cotton. Conversely, under ionized brackish water irrigation, nitrogen application can be reduced to achieve quality and efficiency goals. Cotton growth could be hindered and nitrogen absorption and utilization efficiency may decrease due to a high nitrogen application rate of 450 kg·ha^−1^.

## 5 Conclusion

In 2017, 2018, and 2019, the utilization of 300 kg·ha^−1^ nitrogen application rate during ionized brackish water irrigation consistently led to elevated cotton yield, along with the highest WUE and aNUE. This may be considered the optimal combination of ionized brackish water and nitrogen application pattern for drip-irrigated cotton production in the Xinjiang. More studies are required to determine the ionized brackish water and nitrogen application pattern of drip-irrigated cotton with various soil types.

## Data availability statement

The original contributions presented in the study are included in the article/supplementary material. Further inquiries can be directed to the corresponding author.

## Author contributions

KW: Writing – original draft. QW: Supervision, Writing – review & editing. MD: Supervision, Writing – review & editing. SL: Investigation, Writing – review & editing. YG: Investigation, Writing – review & editing.

## References

[B1] Albornoz F. Lietn J. H. (2015). Over fertilization limits lettuce productivity because of osmotic stress. Chil. J. Agric. Res. 75, 284–290. doi: 10.4067/S0718-58392015000400003

[B2] Assouline S. Russo D. Silber A. Or D. (2015). Balancing water scarcity and quality for sustainable irrigated agriculture. Water Resour. Res. 51, 3419–3436. doi: 10.1002/2015WR017071

[B3] Bondada B. Oosterhuis D. (2001). Canopy photosynthesis, specific leaf weight, and yield components of cotton under varying nitrogen supply. J. Plant Nutr. 24, 469–477. doi: 10.1081/PLN-100104973

[B4] Boquet D. Breitenbeck G. (2000). Nitrogen rate effect on partitioning of nitrogen and dry matter by cotton. Crop Sci. 40, 1685–1693. doi: 10.2135/cropsci2000.4061685x

[B5] Bouksila F. Bahri A. Berndtsson R. Persson M. Rozema J. van der Zee S. (2013). Assessment of soil salinization risks under irrigation with brackish water in semiarid Tunisia. Environ. Exp. Bot. 92, 176–185. doi: 10.1016/j.envexpbot.2012.06.002

[B6] Chen B. Yang H. Song W. Liu C. Xu J. Zhao W.. (2016). Effect of N fertilization rate on soil alkali-hydrolyzable N, subtending leaf N concentration, fiber yield, and quality of cotton. Crop J. 4, 323–330. doi: 10.1016/j.cj.2016.03.006

[B7] Chen W. Hou Z. Wu L. Liang Y. Wei C. (2010). Effects of salinity and nitrogen on cotton growth in arid environment. Plant soil. 326, 61–73. doi: 10.1007/s11104-008-9881-0

[B8] Chen X. Cui Z. Fan M. Vitousek P. Zhao M. Ma W.. (2014). Producing more grain with lower environmental costs. Nat. 514, 486–489. doi: 10.1038/nature13609 25186728

[B9] Dai Y. Liao Z. Lai Z. Bai Z. Zhang F. Li Z.. (2023). Interactive effects of planting pattern, supplementary irrigation and planting density on grain yield, water-nitrogen use efficiency and economic benefit of winter wheat in a semi-humid but drought-prone region of northwest China. Agric. Water Manage. 287, 108438. doi: 10.1016/j.agwat.2023.108438

[B10] Ding X. Tian C. Zhang S. Song J. Zhang F. Mi G.. (2010). Effects of NO_3_ ^–^N on the growth and salinity tolerance of *Tamarix laxa* Willd. Plant Soil. 331, 57–67. doi: 10.1007/s11104-009-0231-7

[B11] Dong H. Li W. Eneji A. Zhang D. (2012). Nitrogen rate and plant density effects on yield and late-season leaf senescence of cotton raised on a saline field. Field Crops Res. 126, 137–144. doi: 10.1016/j.fcr.2011.10.005

[B12] Dong Y. Yang J. Zhao X. Yang S. Mulder J. Dörsch P.. (2022). Seasonal dynamics of soil pH and N transformation as affected by N fertilization in subtropical China: An *in situ* 15N labeling study. Sci. Total Environ. 816, 151596. doi: 10.1016/j.scitotenv.2021.151596 34774948

[B13] Fan L. Yuan Y. Ying Z. Lam S. Liu L. Zhang X.. (2019). Decreasing farm number benefits the mitigation of agricultural non-point source pollution in China. Environ. Sci. pollut. Res. 26, 464–472. doi: 10.1007/s11356-018-3622-6 30406587

[B14] Foley J. Ramankutty N. Brauman K. Cassidy E. Gerber J. Johnston M.. (2011). Solutions for a cultivated planet. Nat. 478, 337–342. doi: 10.1038/nature10452 21993620

[B15] Gang X. Liu H. Peng Y. Yang T. Xi Y. Xu S. (2019). Plastic film mulching combined with nutrient management to improve water use efficiency, production of rain-fed maize and economic returns in semi-arid regions. Field Crop Res. 231, 30–39. doi: 10.1016/j.fcr.2018.11.010

[B16] Gerten D. Heck V. Jägermeyr J. Bodisky B. Fetzer I. Jalava M.. (2020). Feeding ten billion people is possible within four terrestrial planetary boundaries. Nat. Sustain. 3, 200–208. doi: 10.1038/s41893-019-0465-1

[B17] Gong P. Liang L. Zhang Q. (2011). China must reduce fertilizer use too. Nat. 473, 284–285. doi: 10.1038/473284e 21593849

[B18] Guo C. Liu X. He X. (2022). A global meta-analysis of crop yield and agricultural greenhouse gas emissions under nitrogen fertilizer application. Sci. Total Environ. 831, 154982. doi: 10.1016/j.scitotenv.2022.154982 35381236

[B19] Han J. Shi J. Zeng L. Xu J. Wu L. (2015). Effects of nitrogen fertilization on the acidity and salinity of greenhouse soils. Environ. Sci. pollut. Res. 22, 2976–2986. doi: 10.1007/s11356-014-3542-z 25226832

[B20] Hassanzadehdelouei M. Ul-Allah S. Madani A. (2022). Cotton fiber quality response to nitrogen depends on source-sink process, boll growth habit, and weather condition. Ind. Crops Prod. 186, 115279. doi: 10.1016/j.indcrop.2022.115279

[B21] Hou Z. Chen W. Li X. Xiu L. Wu L. (2009). Effects of salinity and fertigation practice on cotton yield and ^15^N recovery. Agric. Water Manage. 96, 1483–1489. doi: 10.1016/j.agwat.2009.04.019

[B22] Hou X. Fan J. Hu W. Zhang F. Yan F. Xiao C.. (2021). Optimal irrigation amount and nitrogen rate improved seed cotton yield while maintaining fiber quality of drip-fertigated cotton in northwest China. Ind. Crops Prod. 170, 113710. doi: 10.1016/j.indcrop.2021.113710

[B23] Iqbal A. Dong Q. Wang X. Gui H. Zhang H. Zhang X.. (2020). Variations in nitrogen metabolism are closely linked with nitrogen uptake and utilization efficiency in cotton genotypes under various nitrogen supplies. Plants (Basel) 9, 250. doi: 10.3390/plants9020250 32075340 PMC7076418

[B24] Isfan D. (1990). Nitrogen physiological efficiency index in some selected spring barley cultivars. J. Plant Nutr. 13, 907–914. doi: 10.1080/01904169009364125

[B25] Jha S. Ramatshaba T. Wang G. Liang Y. Liu H. Gao Y.. (2019). Response of growth, yield and water use efficiency of winter wheat to different irrigation methods and scheduling in North China Plain. Agric. Water Manage. 217, 292–302. doi: 10.1016/j.agwat.2019.03.011

[B26] Kumar R. Pareek N. Kumar U. Javed T. Al-Huqail A. Rathore V.. (2022). Coupling effects of nitrogen and irrigation levels on growth attributes, nitrogen use efficiency, and economics of cotton. Front. Plant Sci. 13, 890181. doi: 10.3389/fpls.2022.890181 35651778 PMC9149569

[B28] Li N. Li J. Tung S. Shi X. Hao X. Shi F.. (2022). Optimal irrigation amount can increase cotton lint yield by improving canopy structure and microenvironment under non-film deep drip irrigation. J. Clean. Prod. 360, 132156. doi: 10.1016/j.jclepro.2022.132156

[B27] Li H. R. Mei X. R. Nangia V. Guo R. Liu Y. Hao W.. (2021). Effects of different nitrogen fertilizers on the yield, water and nitrogen-use efficiencies of drip-fertigated wheat and maize in the North China Plain. Agric. Water Manage. 243, 106474. doi: 10.1016/j.agwat.2020.106474

[B29] Li P. Ren L. (2021). Evaluating the saline water irrigation schemes using a distributed agro-hydrological model. J. Hydrol. 594, 125688. doi: 10.1016/j.jhydrol.2020.125688

[B30] Liu D. Song C. Fang C. Xin Z. Xi J. Lu Y. (2021). A recommended nitrogen application strategy for high crop yield and low environmental pollution at a basin scale. Sci. Total Environ. 792, 148464. doi: 10.1016/j.scitotenv.2021.148464 34465062

[B31] Ma K. Wang Z. Li H. Wang T. Chen R. (2022). Effects of nitrogen application and brackish water irrigation on yield and quality of cotton. Agric. Water Manage. 264, 107512. doi: 10.1016/j.agwat.2022.107512

[B33] Mehta S. Fryar A. Brady R. Morin R. (2000). Modeling regional salinization of the Ogallala aquifer, Southern High Plains, TX, USA. J. Hydrol. 238, 44–64. doi: 10.1016/S0022-1694(00)00314-0

[B34] Munir M. Tahir M. Saleem M. Yaseen M. (2015). Growth, yield and earliness response of cotton to row spacing and nitrogen management. J. Anim. Plant Sci. 25, 729–738.

[B35] Parida A. Mittra B. Das A. Das T. Mohanty P. (2004). High salinity reduces the content of a highly abundant 23-kDa protein of the mangrove *Bruguiera parviflora* . Planta 221, 135–140. doi: 10.1007/s00425-004-1415-2 15580524

[B36] Qi D. Pan C. (2022). Responses of shoot biomass accumulation, distribution, and nitrogen use efficiency of maize to nitrogen application rates under waterlogging. Agric. Water Manage. 261, 107352. doi: 10.1016/j.agwat.2021.107352

[B37] Raphael J. P. A. Echer F. R. Rosolem C. A. (2019). Shading and nitrogen effects on cotton earliness assessed by boll yield distribution. Crop Sci. 59, 697–707. doi: 10.2135/cropsci2018.05.0343

[B38] Ren C. Liu S. Grinsven H. Reis S. Jin S. Liu H.. (2019). The impact of farm size on agricultural sustainability. J. Clean. Prod. 220, 357–367. doi: 10.1016/j.jclepro.2019.02.151

[B39] Ren K. Xu M. Li R. Zheng L. Liu S. Reis S.. (2022). Optimizing nitrogen fertilizer use for more grain and less pollution. J. Clean. Prod. 360, 132180. doi: 10.1016/j.jclepro.2022.132180

[B40] Rinehardt J. Edmisten K. Wells R. Faircloth J. (2004). Response of ultra–narrow and conventional spaced cotton to variable nitrogen rates. J. Plant Nutr. 27, 743–755. doi: 10.1081/PLN-120030379

[B41] Rochester I. (2011). Assessing internal crop nitrogen use efficiency in high-yielding irrigated cotton. Nutr. Cycling Agroecosyst. 90, 147–156. doi: 10.1007/s10705-010-9418-9

[B42] Rodell M. Famiglietti J. Wiese D. Reager J. Beaudoing H. Landerer F.. (2018). Emerging trends in global freshwater availability. Nat. 557, 651–659. doi: 10.1038/s41586-018-0123-1 PMC6077847 29769728

[B5050] Shi F. Li N. Khan A. Lin H. Tian Y. Shi X.. (2022). DPC can inhibit cotton apical dominance and increase seed yield by affecting apical part structure and hormone content[J]. Field Crops Res. 2022, 108509. doi: 10.1016/j.fcr.2022.108509

[B43] Si Z. Zain M. Mehmood F. Wang G. Gao Y. Duan A. (2020). Effects of nitrogen application rate and irrigation regime on growth, yield, and water-nitrogen use efficiency of drip-irrigated winter wheat in the North China Plain. Agric. Water Manage. 231, 106002. doi: 10.1016/j.agwat.2020.106002

[B44] Skaggs T. Anderson R. Corwin D. Suarez D. (2014). Analytical steady-state solutions for water-limited cropping systems using saline irrigation water. Water Resour. Res. 50, 9656–9674. doi: 10.1002/2014WR016058

[B45] Snider J. Harris G. Roberts P. Meeks C. Chastain D. Bange M.. (2021). Cotton physiological and agronomic response to nitrogen application rate. Field Crop Res. 270, 108194. doi: 10.1016/j.fcr.2021.108194

[B46] Stamatiadis S. Tsadilas C. Samaras V. Schepers J. Eskridge K. (2016). Nitrogen uptake and N-use efficiency of Mediterranean cotton under varied deficit irrigation and N fertilization. Eur. J. Agron. 73, 144–151. doi: 10.1016/j.eja.2015.11.013

[B47] Sui B. Feng X. Tian G. Hu X. Shen Q. Guo S. (2013). Optimizing nitrogen supply increases rice yield and nitrogen use efficiency by regulating yield formation factors. Field Crop Res. 150, 99–107. doi: 10.1016/j.fcr.2013.06.012

[B48] Sun L. Li B. Yao M. Zhao M. Niu H. Xu Z.. (2023). Moderate water deficit and nitrogen application rate are conducive to improving the nitrogen uptake and yield of greenhouse tomatoes. Rhizosphere. 28, 100789. doi: 10.1016/j.rhisph.2023.100789

[B49] USDA-NRCS (2020). Natural Resources Conservation Service, United States Department of Agriculture (America: Web Soil Survey). Available at: http://websoilsurvey.sc.egov.usda.gov/.

[B50] Van Dijk M. Morley T. Rau M. Saghai Y. (2021). A meta-analysis of projected global food demand and population at risk of hunger for the period 2010–2050. Nat. Food. 2, 494–501. doi: 10.1038/s43016-021-00322-9 37117684

[B53] Wang S. Mao L. Shi J. Nie J. Song X. Sun X. (2021). Effects of plant density and nitrogen rate on cotton yield and nitrogen use in cotton stubble retaining fields. J. Integr. Agr. 20, 2090–2099. doi: 10.1016/S2095-3119(20)63323-8

[B56] Wang Z. Wang Z. Ma L. Lv X. Meng Y. Zhou Z. (2021). Straw returning coupled with nitrogen fertilization increases canopy photosynthetic capacity, yield and nitrogen use efficiency in cotton. Eur. J. Agron. 126, 126267. doi: 10.1016/j.eja.2021.126267

[B54] Wang X. Wang H. Si Z. Gao Y. Duan A. (2020). Modelling responses of cotton growth and yield to pre-planting soil moisture with the CROPGRO-Cotton model for a mulched drip irrigation system in the Tarim Basin. Agric. Water Manage. 241, 106378. doi: 10.1016/j.agwat.2020.106378

[B51] Wang H. Wu L. Cheng M. Fan J. Zhang F. Zou Y.. (2018). Coupling effects of water and fertilizer on yield, water and fertilizer use efficiency of drip-fertigated cotton in Northern Xinjiang, China. Field Crop Res. 219, 169–179. doi: 10.1016/j.fcr.2018.02.002

[B52] Wang Q. Zhang J. Men Q. Tan S. Zhou L. Liu X. (2016). Experiment on physical and chemical characteristics of activated brackish water by magnetization or ionization. Chin. Soc Agric. Eng. 32, 60–66.

[B57] Wei K. Zhang J. Wang Q. Guo Y. Mu W. (2022). Irrigation with ionized brackish water affects cotton yield and water use efficiency. Ind. Crops Prod. 175, 114244. doi: 10.1016/j.indcrop.2021.114244

[B58] Xu M. Zhang Y. Wang Y. Wang L. Bai Y. Lu Y. (2023). Optimizing nitrogen input and nitrogen use efficiency through soil nitrogen balance in a long-term winter wheat-summer maize rotation system in North China. Eur. J. Agron. 149, 126908. doi: 10.1016/j.eja.2023.126908

[B59] Yang G. Li F. Tian L. He X. Gao Y. Wang Z.. (2020). Soil physicochemical properties and cotton (*Gossypium hirsutum* L.) yield under brackish water mulched drip irrigation. Soil Til. Res. 199, 104592.

[B60] Yang X. Ni K. Shi Y. Yi X. Zhang Q. Fang L.. (2018). Effects of long-term nitrogen application on soil acidification and solution chemistry of a tea plantation in China. Agric. Ecosyst. Environ. 252, 74–82. doi: 10.1016/j.agee.2017.10.004

[B61] Zhang D. Li W. Xin C. Tang W. Eneji A. Dong H. (2012). Lint yield and nitrogen use efficiency of field-grown cotton vary with soil salinity and nitrogen application rate. Field Crop Res. 138, 63–70. doi: 10.1016/j.fcr.2012.09.013

[B62] Zhang Y. Wang H. Lei Q. Luo J. Lindsey S. Zhang J.. (2018). Optimizing the nitrogen application rate for maize and wheat based on yield and environment on the Northern China Plain. Sci. Total Environ. 618, 1173–1183. doi: 10.1016/j.scitotenv.2017.09.183 29054672

[B63] Zhang Y. Xun J. Zhang J. Zhang G. Zhang W. Wang K.. (2021). Does nitrogen application rate affect the moisture content of corn grains? J. Integr. Agric. 20, 2627–2638. doi: 10.1016/S2095-3119(20)63401-3

[B64] Zhao G. Mu Y. Wang Y. Wang L. (2021). Response of winter-wheat grain yield and water-use efficiency to irrigation with activated water on Guan Zhong Plain in China. Irrig. Sci. 39, 1–14. doi: 10.1007/s00271-020-00706-y

[B65] Zhou X. Wang R. Gao F. Xiao H. Xu H. Wang D. (2019). Apple and maize physiological characteristics and water-use efficiency in an alley cropping system under water and fertilizer coupling in Loess Plateau, China. Agric. Water Manage 221, 1–12. doi: 10.1016/j.agwat.2019.04.019

